# Supplementary material for: Adequacy of Disease Control by Supraomohyoid Neck Dissection in cT1/T2 Tongue Cancer
Source: J Pers Med. 2022 Sep 19;12(9):1535. doi: 10.3390/jpm12091535 (PMC9505271; doi:10.3390/jpm12091535)
Supplement: Supplementary file 1 [file jpm-12-01535-s001.zip › jpm-1912654-supplementary.pdf]

Supplementary Table S1. Patients' characteristics (*n*= 565).

**Characteristics**		**No. of patients [*n*(%)]**
Age	Mean	49.73
Sex	Male	485 (85.8)
	Female	80 (14.2)
Alcohol drinking	Yes	375 (66.4)
	No	190 (33.6)
Smoking	Yes	408 (72.2)
	No	150 (26.5)
	Missing	7 (1.2)
Betel	Yes	394 (69.7)
	No	171 (30.3)

Supplementary Table S2. Lymph node level involved in patients received modified radical neck dissection (mRND).

**Level**	**pN+ patients**
I	107 (58.1%)
II	101 (55.9%)
III	49 (26.6%)
IV	6 (9.3%)*
V	1 (1.5%)*
Skip metastasis	0%

*Calculated only in mRND group
